# Comparing tuberculosis symptom screening to chest X-ray with artificial intelligence in an active case finding campaign in Northeast Nigeria

**DOI:** 10.1186/s44263-023-00017-2

**Published:** 2023-10-06

**Authors:** Stephen John, Suraj Abdulkarim, Salisu Usman, Md. Toufiq Rahman, Jacob Creswell

**Affiliations:** 1 Janna Health Foundation, Yola, Adamawa State Nigeria; 2 SUFABEL Community Development Initiative, Gombe, Gombe State Nigeria; 3 Yamaltu Deba, Primary Health Care Department, Gombe, Gombe State Nigeria; 4 Innovations & Grants, Stop TB Partnership, Global Health Campus – Chemin du Pommier 40, Le Grand-Saconnex, Geneva, 1218 Switzerland

**Keywords:** Chest X-ray, Ultra-portable, Artificial intelligence, Tuberculosis, Nigeria, Active case finding

## Abstract

**Background:**

Ultra-portable X-ray devices with artificial intelligence (AI) are increasingly used to screen for tuberculosis (TB). Few studies have documented their performance. We aimed to evaluate the performance of chest X-ray (CXR) and symptom screening for active case finding of TB among remote populations using ultra-portable X-ray and AI.

**Methods:**

We organized screening camps in rural northeast Nigeria, and all consenting individuals ≥ 15 years were screened for TB symptoms (cough, fever, night sweats, and weight loss) and received a CXR. We used a MinXray Impact system interpreted by AI (qXR V3), which is a wireless setup and can be run without electricity. We collected sputum samples from individuals with an qXR abnormality score of 0.30 or higher or if they reported any TB symptoms. Samples were tested with Xpert MTB/RIF. We documented the TB screening cascade and evaluated the performance of screening with different combinations of symptoms and CXR interpreted by AI.

**Results:**

We screened 5297 individuals during 66 camps: 2684 (51%) were females, and 2613 (49%) were males. Using ≥ 2 weeks of cough to define presumptive TB, 1056 people (20%) would be identified. If a cough of any duration was used, the number with presumptive TB increased to 1889 (36%) and to 3083 (58%) if any of the four symptoms were used. Overall, 769 (14.5%) had abnormality scores of 0.3 or higher, and 447 (8.4%) had a score of 0.5 or higher. We collected 1021 samples for Xpert testing and detected 85 (8%) individuals with TB. Screening for prolonged cough only identified 40% of people with TB. Any symptom detected 90.6% of people with TB, but specificity was 11.4%. Using an AI abnormality score of 0.50 identified 89.4% of people with TB with a specificity of 62.8%.

**Conclusions:**

Ultra-portable CXR can be used to provide more efficient TB screening in hard-to-reach areas. Symptom screening missed large proportions of people with bacteriologically confirmed TB. Employing AI to read CXR can improve triaging when human readers are unavailable and can save expensive diagnostic testing costs.

## Background

In 2021, more than 4 million people with tuberculosis (TB) were missed by national TB programs globally [1]. This is often because people have had difficulty accessing health services [2]. When people with TB do access health services, the facility frequently does not have TB diagnostic capabilities [3]. People with TB can also be missed by poor screening [4] or insensitive diagnostic tools [5]. Mobile chest X-ray (CXR) in active case finding (ACF) campaigns to identify people with TB were widely used in the middle of the last century but fell out of favor after the World Health Organization (WHO) recommended against the practice [6]. More recently, however, CXR has been increasingly used to improve the sensitivity of TB screening approaches. The introduction of newer X-ray technology, less expensive equipment, and the findings of modern TB prevalence survey’s which demonstrate the ability of CXR to detect asymptomatic people with TB have led to a renewed interest in mobile campaigns to screen people for TB [7–10]. CXR can improve sensitivity of the TB algorithm by identifying people who have TB but would be missed by symptom screening alone [5, 11, 12]. Additionally, it can reduce the number of diagnostic tests needed because people with normal lung findings are unlikely to have pulmonary TB [13, 14]. CXR is also an important tool for diagnosing pulmonary TB in the absence of bacteriological evidence [15].

Nigeria has the highest TB burden in Africa, 5th highest globally, and despite impressive gains in TB treatment coverage in the last few years, 56% of people developing TB in 2021 were still missed [1]. Reasons for the gaps in TB care are numerous, but lack of access to diagnostic services, especially WHO-endorsed modern molecular tests like Xpert MTB/RIF (Xpert) testing which enables the simultaneous detection of TB and resistance to rifampicin in 2 h [16], and CXR, both contribute. In northeast Nigeria, poverty, political instability and corruption, and domestic terrorism from Boko Haram all lead to weak public health infrastructure and poor access to care [17]. In Adamawa state, there were only five functioning X-ray machines in public sector facilities in 2022 and only two trained radiologists working in the public sector for an estimated population of over 4.5 million. These factors contributed to TB notification rates of only 89/100,000 in Adamawa, while the TB burden estimate for Nigeria is 219/100,000 [18].

While modern X-ray machines have been mounted into mobile units as part of community outreach for TB [10, 19, 20], some populations remain difficult to access even by these trucks. Recently, ultraportable X-ray devices, which can be more easily transported in a small vehicle, have been deployed [7, 21]. These machines are equipped with artificial intelligence (AI) to interpret the CXR image and assist the staff in deciding whether or not to request a sample for diagnostic testing. Because ultra-portable X-ray machines are relatively new, published evaluations around how to employ them and their potential impact on algorithm performance for TB detection have been confined to case studies and gray literature [21]. Here, we report on the results of a cross-sectional study using CXR and AI as part of TB screening in remote rural Nomadic populations of northeast Nigeria. Our aim was to evaluate the performance of different combinations of symptom screening and CXR using ultra-portable X-ray and AI to identify people with bacteriologically confirmed TB in remote populations.

## Methods

The study was carried out in 15 local government areas (LGA, similar to sub-districts) in two states, Gombe and Adamawa, in northeast Nigeria. There are 32 LGAs in the two states, and the 15 were selected purposefully with state administrators as they have a higher concentration of nomadic communities. A series of community camps were implemented from 17 July until 6 December 2022. Camps were held in schools, primary health facilities, and gathering points after consultations with local leaders and LGA supervisors to identify communities with limited access to health services. A mobile team consisting of a registration officer, data entry staff, a radiographer, and a coordination officer attended each community camp. Prior to the event, permissions were obtained from community leaders to promote attendance, and coordination with the nearest Xpert testing facility was undertaken to ensure sufficient cartridge capacity would be available. The camps were promoted as lung health camps to encourage a wide variety of attendees.

We used a MinXray (Northbrook, IL, USA) Impact system consisting of a TR90BH battery-powered portable X-ray generator with a wireless cesium iodide detector plate, a lead apron for the radiographer, and a laptop equipped with qXR version 3 (Qure.ai Mumbai, India) to conduct the CXR screenings. qXR is an AI software that interprets CXR images for TB abnormalities and has been recommended by WHO to use for TB screening and triage [22]. The qXR algorithm interprets the image and provides a TB abnormality score ranging from 0.01 to 0.99 in a few seconds. The abnormality score is calculated by the AI algorithm based on the extent and distribution of radiological patterns (presence of cavity, nodules, etc.) consistent with TB. The cumulative score results from each identified pattern, and the higher the score, the greater the radiological involvement. The X-ray machine and laptop were packed into two suitcases for transport. The AI interpreted all CXR images immediately onsite. During the community camps, all individuals older than 15 who were not currently receiving TB treatment were eligible to participate in TB screening. Individuals were provided verbal consent to participate and receive the screening tests. All consenting participants were screened for TB symptoms verbally (cough and duration, fever, night sweats, and weight loss) and offered a CXR.

After symptom and radiological screening, those individuals with a TB abnormality score of 0.30 or more on qXR and/or reporting one or more TB symptoms were requested to produce a sputum sample. Samples were transported to the nearest Xpert testing facility using a cold box. Test results were retrieved by study staff the following day and returned to the person with presumptive TB. Anyone with a positive Xpert result was initiated on anti-TB treatment as per national TB guidelines.

Qure.ai suggests a threshold score of 0.5 when using qXR. Several evaluations of qXR have shown variable ideal abnormality thresholds [23–25]. We chose to reduce the threshold score to 0.3 to improve the sensitivity and capture more people with presumptive TB to analyze the performance of CXR screening. We could not test asymptomatic individuals regardless of their abnormality score due to resource constraints.

Data on basic demographics including age and sex, as well as symptoms and screening and testing results, were collected in a Microsoft Excel workbook during the community camps. All CXR’s and the corresponding AI data were captured using Qure.ai’s qTrack software which houses CXR images and offers remote access to study coordinators. We documented the screening cascade and evaluated the performance of CXR, symptoms alone, and combined (CXR and symptoms) screening. We used three verbal screening modalities to define someone with presumptive TB: (1) the traditional TB screening cough for 2 weeks or more, (2) any cough, and (3) any symptom. To evaluate the performance of the different screening strategies, we modeled the potential to save Xpert cartridge testing by using CXR as a screening tool compared to different types of symptom screening. All analyses were conducted using the Statistical Package for Social Sciences, version 26 (SPSS Inc., Chicago, IL, USA).

## Results

**Fig. 1:**
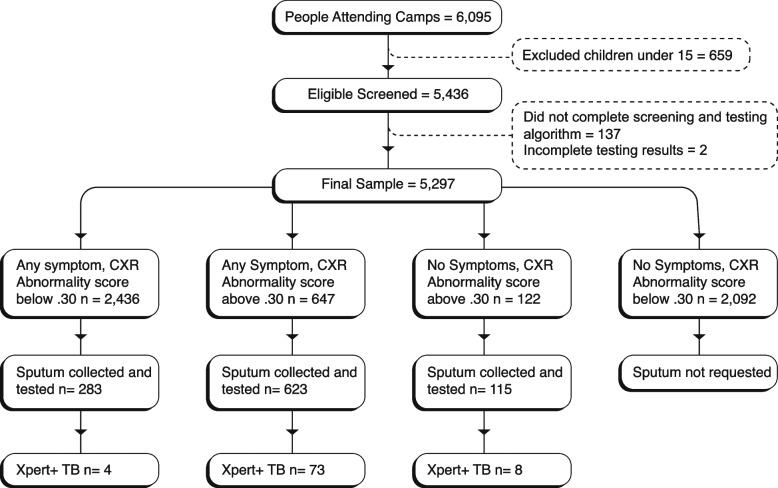
Study flow chart

During the study period, 66 screening events were held in 60 different communities. There were 5436 eligible people attending, and we enrolled 5297 individuals aged 15 and above who completed the verbal symptoms and CXR screening with valid results. Overall, 3205 people reported either any TB symptom or had an AI abnormality score of 0.30 or higher, while 2092 individuals had reported no symptoms and had normal CXR images (Fig. 1). We screened 2684 (51%) females and 2613 (49%) males (Table 1). The median age of males was 39 (*IQR*: 26–53), and for females, it was 36 (*IQR*: 26–48). The different verbal symptom screening approaches yielded varied results. Using the traditional prolonged cough (2 weeks or more), 1056 people (20%) had presumptive TB. If cough of any duration was used, the number with presumptive TB increased to 1889 (36%) and to 3083 (58%) if any of the four symptoms were used. Overall, 769 participants (14.5%) had abnormality scores of 0.3 or higher, and 447 (8.4%) had a score of 0.5 or higher. We were able to collect 1021 samples for Xpert testing with sputum collection rates varying depending on symptoms and abnormality scores. While sputum collection among people with any TB symptom was low, 29% (906 of 3083), it was 96% (738 of 769) among people with AI scores 0.3 and above and 96% (623 of 647) of people with AI scores of 0.3 and above and reporting any TB symptom. Xpert testing detected 85 people (8% of those tested) with TB (1604/100,000 screened), all of whom were linked to treatment. Of these, we identified 53 men (2028/100,000 screened) and 36 women (1341/100,000 screened). None of the 85 individuals had rifampicin-resistant results.

**Table 1:** Demographic and clinical description of participants with AI abnormality scores among different groups

**All participants** **5297**	***n* (%)**	**Median score (IQR)**
**Gender**		
Male	2613 (49%)	0.037 (0.018–0.13)
Female	2684 (51%)	0.042 (0.019–0.14)
**Age, median (IQR)**	37 (26–50)	
Male, median (IQR)	39 (26–53)	-
Female, median (IQR)	36 (26–48)	-
15–24	1054 (20%)	0.035 (0.018–0.086)
25–50	2950 (56%)	0.039 (0.018–0.13)
> 50	1293 (24%)	0.048 (0.020–0.196)
**TB symptoms**		
Any cough	1889 (36%)	0.05 (0.022–0.25)
Cough ≥ 2 weeks	1056 (20%)	0.05 (0.022–0.265)
Fever	425 (8%)	0.132 (0.028–0.51)
Weight loss	1589 (30%)	0.048 (0.022–0.194)
Night sweats	1403 (26%)	0.045 (0.021–0.178)
Any TB symptoms	3083 (58%)	0.05 (0.021–0.21)
**TB risk factors**		
History of smoking	561 (11%)	0.052 (0.021–0.24)
HIV positive (self-reported)	32 (0.6%)	0.134 (0.046–0.95)
**Sputum collection**	1021 (32% of 3205)	0.43 (0.24–0.74)
Any cough	639 (34% of 1889)	0.40 (0.096–0.68)
Cough ≥ 2 weeks	394 (37% of 1056)	0.36 (0.086–0.67)
Fever	212 (50% of 425)	0.49 (0.034–0.85)
Weight loss	515 (32% of 1589)	0.41 (0.013–0.71)
Night sweats	430 (31% of 1403)	0.38 (0.098–0.68)
Any TB symptoms	906 (29% of 3083)	0.43 (0.02–0.72)
History of smoking	174 (33% of 561)	0.44 (0.019–0.84)
AI score 30 and above	738 (96% of 769)	-
AI score 50 and above	424 (95% of 447)	-
AI score ≥ 30 AND any symptom	623 (96% of 647)	-
**Xpert MTB/RIF results**		
Test performed	1021 (19%)	
MTB detected	85 (8%)	0.91 (0.67–0.97)
MTB not detected	936 (92%)	0.42 (0.21–0.65)

*IQR* interquartile range, *TB* tuberculosis, *HIV* human immunodeficiency virus, *AI* artificial intelligence, *MTB Mycobacterium tuberculosis*, *RIF* rifampicin

Overall, the median abnormality score on qXR was 0.04 (*IQR*: 0.02–0.14) and increased as people got older (0.035 among those under 25, 0.039 from 25 to 50 and 0.048 in people above 50). There were little differences in the median abnormality score between men (0.037) and women (0.042). Among people with any symptoms, the mean abnormality score was 0.05 (*IQR*: 0.021–0.21) increasing to 0.91 (*IQR*: 0.67–0.97) among people with symptoms and TB. Among all people tested with Xpert, the mean abnormality score for people with and without TB was 0.91 and 0.42.

The distribution of abnormality scores on qXR was heavily skewed left with 4528 people (85.5%) having scores below 0.30 (Table 2). There were 158 (3%) with scores 0.9 or above, and this group accounted for 53% (45 of 85) of people with TB detected on Xpert. Among people with abnormality scores at or above 0.3, Xpert positivity was 11%. However, Xpert yields were below 2% for abnormality scores between 0.3 and 0.5, while they were 17.7% at 0.5 and above and 28.7% at 0.9 and above.

**Table 2:** GeneXpert yield in different AI band scores

**AI abnormality score**	**Count**	**Tested** ***n* (% of count)**	**MTB detected** ***n* (% of tested)**
0.90–0.99	158	157 (99%)	45 (28.7%)
0.80–0.89	94	72 (77%)	14 (19%)
0.70–0.79	45	45 (100%)	5 (11%)
0.60–0.69	62	62 (100%)	3 (5%)
0.50–0.59	94	94 (100%)	9 (10%)
0.40–0.49	154	153 (99%)	3 (2%)
0.30–0.39	162	155 (96%)	2 (1%)
0.20–0.29	247	59 (24%)	2 (3%)
0.10–0.19	583	46 (8%)	1 (2%)
0–0.09	3698	178 (5%)	1 (0.6%)

*AI* artificial intelligence, *MTB Mycobacterium tuberculosis*

Table 3 shows the performance of different screening combinations of symptoms and CXR with AI. Overall, we conducted 1021 Xpert tests to detect 85 people with bacteriologically positive TB. Screening using *cough of 2 weeks or more* had a sensitivity of 40% and a specificity of 61.5%, using 394 tests. Using *any cough* to screen would have identified 53 individuals with TB (62.4% sensitivity) and used 639 tests. If *any symptom* was used to screen, then the sensitivity rose to 90.6% (77 of the 85 people with TB would be identified), but 906 tests would be needed. Using CXR alone with a threshold score of 0.30 produced a sensitivity of 95.3% and a specificity of 29.8%, requiring 738 Xpert cartridges. Using a higher cutoff of 0.50 only used 424 tests to identify 89.4% of people with TB with a specificity of 62.8%. Using a combined screening approach including CXR with a threshold score at or above 0.5 with cough of 2 weeks or more identified all but two people with TB on Xpert and used 682 cartridges.

**Table 3:** Yield of different algorithms in TB detection and utilization of tests

**Algorithm**	**Sensitivity**	**Specificity**	**PPV**	**NPV**	**People with TB missed**	**Tests used**
Abnormality ≥ 0.30	95.3%	29.8%	16.9%	97.7%	4	738
Abnormality ≥ 0.50	89.4%	62.8%	26.4%	97.5%	9	424
Any cough	62.4%	37.4%	12.9%	86.9%	32	639
Cough ≥ 2 weeks	40%	61.5%	13.5%	87.3%	51	394
Any symptom	90.6%	11.4%	13.3%	89.1%	8	906
Cough OR fever	67.1%	29.7%	12.5%	85.8%	28	715
Abnormality ≥ 0.30 OR any cough	100%	5.9%	13.7%	100%	0	966
Abnormality ≥ 0.30 OR cough ≥ 2 weeks	100%	13.4%	14.7%	100%	0	896
Abnormality ≥ 0.30 OR any symptom	100%	0%	13%	-	0	1021
Abnormality ≥ 0.50 OR any cough	100%	20.5%	15.8%	100%	0	829
Abnormality ≥ 0.50 OR cough ≥ 2 weeks	97.7%	36%	18.6%	99%	2	682
Abnormality ≥ 0.50 OR any symptom	100%	5.7%	13.7%	100%	0	968

*PPV* positive predictive value, *NPV* negative predictive value

## Discussion

Our results add to a small body of evidence on the use of ultra-portable X-ray devices for screening for TB in a community-based setting. We found that ultra-portable CXR can be used to reach large numbers of people in remote rural Nigeria without electricity and provide improved sensitivity and specificity compared to traditional symptom screening approaches. The use of CXR with AI in addition to symptom screening greatly improved sensitivity, increasing 60% over traditional screening for prolonged cough. Moreover, CXR with a qXR version 3 AI threshold of 0.5 alone identified almost the same number of people with TB as testing people reporting any symptom, but CXR with AI screening saved more than 50% of the tests needed and came the closest to meeting WHO target product profile targets of 90% sensitivity and 70% specificity [26]. Odume et al. recently conducted ACF in Nigeria using a different ultra-portable X-ray system, but did not compare yields of verbal and CXR screening approaches [27]. They screened a higher number of people in a 5-month period and identified 1.2% of the population with TB, 40% of whom were bacteriologically confirmed. Our results only focused on individuals with bacteriologically confirmed TB, yielding 1.6% among those screened. A study from Vietnam reported on different aspects on the performance of an ultra-portable X-ray device, mostly focused on image assessment and radiation levels [7]. The new ultra-portable devices are much lighter and can be transported by car, set up in minutes, and used without physicians or radiologists onsite, thus expanding reach to areas with poor access to healthcare. More research is needed on how these ultra-portable machines can be optimized and the potential impact in different populations, settings, and the costs to do so.

In our study, 10% of people with bacteriologically confirmed TB on Xpert did not report any TB symptom, and almost 40% did not report cough. Nigeria’s TB prevalence survey demonstrated the added value of using CXR to identify people who do not complain of symptoms but have bacteriologically confirmed TB. Thirty-six percent of people with bacteriologically confirmed TB in the prevalence survey did not report symptoms [28]. An ACF study from India showed similar results among household contacts of people with TB [5]. An earlier study from Vietnam demonstrated that 50% of people would be missed by using symptoms alone [11]. Both of these studies used fixed X-ray systems where people had to travel to health facilities to be tested. Barriers to access to CXR can erode the improvements in sensitivity achieved by using CXR in screening [29]. Conducting ACF within communities can reduce access barriers, identify more people with TB, and reduce out of pocket costs [30–32]; more evaluations with new technology could improve our understanding of how screening with CXR and AI works. A recent systematic review evaluating TB symptoms screening and CXR showed that most studies including both symptoms and CXR screening were done in TB prevalence surveys or prisons, and they did not include AI [33], although a recent study from South Africa using mobile van screening documented almost 4 in 5 people with TB were asymptomatic [34], far more than in our results.

Compared to symptom screening for any TB symptom, using CXR and qXR V3 with a threshold of 0.3 produced a 5% improvement in sensitivity and used 23% fewer tests. If a threshold of 0.5 was used, the sensitivity loss would be 1%, but less than half of the Xpert tests would be used creating a savings of almost US $5000 in test costs alone. Studies in Bangladesh, Pakistan, and South Africa documented potential savings of Xpert testing using CXR and AI [13, 35, 36]. The clear gradient of Xpert positivity by threshold scores potentially provides an additional way to save Xpert cartridge costs. During the Covid-19 pandemic, several studies demonstrated the promise of pooling sputum to reduce the number of tests and time needed to diagnose someone with TB [37–39]. Using the abnormality score could help further reduce testing requirements if, for example, those with scores above 0.70 were tested individually, but lower scores were pooled since the positivity rates were much lower.

While using the ultra-portable X-ray machines with AI was a more efficient screening tool that symptoms and identified people with TB missed by traditional symptom screening, the interest generated from the camps is also worth mentioning. There was large demand at the camps for the screenings, with some members requesting a second CXR when they perceived their AI score was not high. Acceptance for the screening was 100%, and community leaders requested more days than were available for lung health screening. Although we could not measure it analytically, bringing this technology to the community level clearly helped participation as word spread about the machines and what they can do. It should be noted that there were some important observations about using the technology. The initial trainings were done onsite which helped build local capacity, and the X-ray generator worked very well. Minor issues around were easily addressed with remote support from the manufacturer. Although the X-ray machine came with a spare battery which was critical, we needed to procure a second backup for the laptop which would have run out of life during long days. The software worked without any wires and read the images quickly so results could be returned and sputum requested immediately, but using qTrack’s flexibility to configure demographic data with dedicated fields (i.e., for specific symptoms) would have reduced data entry work.

Our findings have several limitations. We were not fully able to evaluate the performance of the AI software as we did not test participants with abnormality scores below 0.30, likely missing some people with TB. However, the positivity rate on Xpert for those with abnormality scores from below 0.5 was less than 3% even among people with symptoms, so we do not expect many people with TB who had low AI scores and no symptoms to have been missed. Neither the communities nor participants were randomly selected, and they do not represent levels of TB in the communities they came from. The camps were promoted as lung health screenings, and people with lung problems were encouraged to attend. In ACF approaches, indiscriminate screening is not recommended, so some form of self-selection is expected. While we calculated the number of tests saved, we did not include an analysis of the cost of the CXR machine and AI. Running costs of the machine are not high, but upfront costs are substantial, so doing a lot of TB screening would improve cost per person screened, particularly as AI companies offer unlimited packages as well. The machines can also be used for non-TB-related work, increasing their utility, which is also increasingly the case with AI. Finally, the sensitivity of a single Xpert test is much less than 100%, and therefore, some people with TB were missed who could have been diagnosed clinically. It is not feasible to have access to radiologists in these remote areas for many screening events, but remote reading can identify more people with TB and link them to care.

## Conclusions

Symptoms screening for TB misses many people with bacteriologically confirmed TB, even with multiple screening questions. Using ultra-portable CXR equipped with AI identified many more people with TB and reduced testing requirements in rural Nigeria, could be used without electricity, and created high interest from community members. Employing AI to read CXR can improve triaging when human readers are not available and was simple to implement by health workers with basic training. More sensitive and specific algorithms using these lightweight devices can help identify more people with TB and link them to treatment.

## Data Availability

The data that support the findings of this study can be made available with the permission of Adamawa State Ministry of Health and Gombe State Ministry of Health through a request to admoh_yola@yahoo.com.
